# Concentration and purification by magnetic separation of the erythrocytic stages of all human *Plasmodium *species

**DOI:** 10.1186/1475-2875-7-45

**Published:** 2008-03-05

**Authors:** Clotilde Ribaut, Antoine Berry, Séverine Chevalley, Karine Reybier, Isabelle Morlais, Daniel Parzy, Françoise Nepveu, Françoise Benoit-Vical, Alexis Valentin

**Affiliations:** 1 Université de Toulouse, Laboratoire Pharmacochimie des Substances Naturelles et Pharmacophores Redox, UMR 152 IRD-Université, Université Paul Sabatier, Faculté de Pharmacie, 118 route de Narbonne, 31062 Toulouse Cedex 9, France; 2 Université de Toulouse, Service de Parasitologie et Mycologie, Hôpital Rangueil-Larrey CHU de Toulouse et Faculté de Médecine de Rangueil, 1 av. Jean Poulhes – TSA 50032, 31059 Toulouse cedex 9, France; 3 Laboratoire d'entomologie médicale, OCEAC-IRD, BP288, Yaoundé, Cameroon; 4 Unité de Parasitologie, IMTSSA, 13998 Marseille Armées, France; 5 Laboratoire de Chimie de Coordination du CNRS, UPR8241, 31077 Toulouse 4, France

## Abstract

**Background:**

Parasite concentration methods facilitate molecular, biochemical and immunological research on the erythrocytic stages of *Plasmodium*. In this paper, an adaptation of magnetic MACS^® ^columns for the purification of human *Plasmodium *species is presented. This method was useful for the concentration/purification of either schizonts or gametocytes.

**Results and conclusions:**

The magnetic removal of non-parasitized red blood cells (in vivo and in vitro) using magnetic columns (MACS) was evaluated. This easy-to-use technique enriched schizonts and gametocytes from *Plasmodium falciparum *in vitro cultures with a very high degree of purity. In addition, all haemozoin-containing stages (schizonts and/or gametocytes) from the peripheral blood of infected patients could be concentrated using this method. This method is particularly useful for the concentration of non-falciparum species, which do not grow in culture and are otherwise difficult to obtain in large amounts.

## Background

Falciparum malaria is one of the most common diseases in tropical countries. There are about 300 million new cases, and more than two million deaths due to this illness every year [1]. Different methods have been described to isolate and/or purify living parasites from in vitro cultures or from murine *Plasmodium *[2,3]. However, non-falciparum species of human *Plasmodium *(namely *Plasmodium vivax*, *Plasmodium ovale*, and *Plasmodium malariae*) are not easy to purify or concentrate from the blood of infected patients. Difficulties in producing large amounts of parasite material from the blood stages of *Plasmodium sp. *have restricted progress in research on non-falciparum malaria, but also studies on gametocyte biology. Moreover, in endemic regions, the use of an easy single-step method for the purification and/or concentration of the gametocytes could be useful for biological and epidemiological studies [4]. The property of all human *Plasmodium *species to degrade haemoglobin (an Fe(II) diamagnetic complex) into haemozoin (an Fe(III) paramagnetic complex) was used, making possible the magnetic purification of parasitized red blood cells containing haemozoin. The present study demonstrates the high degree of purity that can be obtained for the synchronization of in vitro cultures of *Plasmodium falciparum*, either on asexual or sexual erythrocytic stages, and the usefulness of magnetic separation for the enrichment and purification of *Plasmodium *parasitized red blood cells from infected malaria patients.

## Methods

### In vitro culture of *Plasmodium falciparum*

Parasites were cultured according to the method described by Trager and Jensen [5], with modifications [6]. Briefly, parasites were maintained in human erythrocytes (O ±, Blood Bank, EFS, Toulouse, France) routinely at 0.5–4% parasitaemia in culture medium (haematocrit: 2–4%). The culture medium consisted of RPMI (Cambrex, Belgium) complemented with 25 mM Hepes (Cambrex) and 2 mM glutamine (Sigma, l'Isle d'Abeau, France) and supplemented with 7.5% human serum (EFS). The knob+ strains (FcB1-Colombia, W2-Indochina and F32-Tanzania) were concentrated by flotation with Plasmion^® ^(Fresenius Kabi France) followed by 5% D-sorbitol (Sigma) lysis [2]. The knobby-strain (FcM29-Cameroon) was only synchronized by 5% D-sorbitol lysis every 48 hrs [7].

Gametocyte cultures of strain W2 were initiated as described elsewhere [8], with minor modifications [9]. Cultures were then treated with 50 mM N-acetyl-D-glucosamine (Sigma) for 3–5 days to remove most of the asexual stages. Young (stage II, 7-day-old) or old (stage IV–V, 13-day-old) gametocyte cultures were tested for magnetic enrichment.

### Parasitized blood from patients

Parasitized blood from patients presenting symptoms of malaria was obtained by venipuncture for diagnosis purposes in the University Hospital in Toulouse. One EDTA-tube was routinely filled. Diagnosis (malaria, *Plasmodium *species and parasitaemia) was made by QBC^® ^associated to thin smear and the *Plasmodium *species and single infection was confirmed by routine real-time PCR [10]. When a non-falciparum malaria was diagnosed or when gametocytes (whatever the *Plasmodium *species) were found, the remaining blood was used for the concentration of the parasites with magnetic columns. Before passage on columns, the blood cells were washed (centrifugation at 800 g, 10 min) once in 10 volumes of warmed culture PBS (Cambrex). The enrichment of *P. falciparum *gametocytes was also carried out on blood from Cameroonian patients with diagnosed malaria (IRD-OCEAC, Yaounde, Cameroon). In every case, the blood was washed once with PBS without human serum before passage on the columns.

Each sample from Toulouse University Hospital patients corresponded to an EDTA-tube bottom that was used for malaria diagnosis. Experiments did not need any additional blood sample. Blood samples were anonymized and any remaining material was discarded in order to respect the confidentiality of participants and protection of personal data. Protocol for gametocyte carrier recruitment received ethical approval from the National Ethic Committee in Cameroon (039/CNE/MP/06).

For both sampling sites (France and Cameroon), trophozoite parasitaemia was evaluated on thin smears while a gametocyte count was performed on calibrated 5 μL thick films before the enrichment was carried out. For some falciparum-infected patients from Cameroon, blood samples, with no evidence for the presence of gametocytes on thick films were also treated with magnetic columns for gametocyte enrichment.

### Purification

Whether from culture or from patients, parasitaemia in infected erythrocytes was evaluated by two experienced technicians before any experimentation. Prior to purification, the MACS^® ^(25 LD columns, Miltenyi Biotec, Germany) columns were filled with warmed (37°C) RPMI or PBS. When the purification was carried out in order to synchronize the culture, the experimentation was performed under sterile conditions. The test blood (from culture or from patients) was then deposited on the top of the column (typically, 1 mL at 25–50% haematocrit) which was held in a Quadro MACS^® ^magnetic support. Warmed (37°C) culture medium was then added until the eluent was apparently free of red blood cells. The column was removed from the magnetic support and a further 4 mL (dead volume of the column) of culture medium added and the eluent recovered. This eluent was then centrifuged (800 g, 10 min) and the supernatant was discarded. The pellet was used to prepare blood smears and calibrated (5 μL) thick films, both stained with Giemsa.

All images were digitally registered with a Nikon digital camera (DXM1200) mounted on a Nikon microscope (Eclipse E 400) and not modified with any software. Visual counting of gametocytes was performed on Giemsa-stained smears before and after concentration.

## Results and discussions

### Concentration and synchronization of *P. falciparum *schizonts

**Figure 1:**
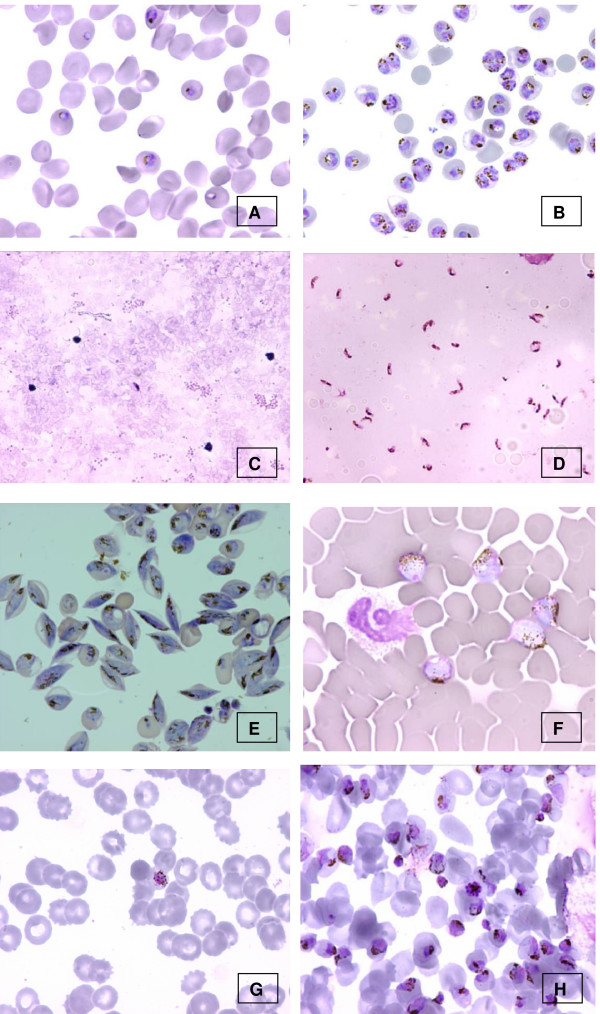
*Plasmodium *purification with MACS^® ^(magnification ×100, except C and D: × 40) *P. falciparum *in vitro culture before (A) and after (B) purification (thin smears). *Plasmodium falciparum *gametocyte containing blood before (C) and after (D) purification (calibrated thick film). *Plasmodium falciparum *gametocytes obtained from in vitro culture (E). *Plasmodium ovale *gametocytes from an infected patient (F). *Plasmodium malariae *schizonts before (G) and after (H) purification (thin smears)

As *P. falciparum *schizonts contained paramagnetic Fe(III) species, they were highly purified by the MACS^® ^columns. Table 1 summarizes the results obtained for *P. falciparum *cultured in vitro. Clearly, MACS^® ^enabled *P. falciparum *infected erythrocytes to be purified when containing paramagnetic iron(III) species (Figures 1A and 1B). This synchronization was efficient either with knobby (K+) or with knobless (K-) strains while and, as expected, only K+ strains were enriched with Plasmion^®^. The yield of purity after magnetic enrichment was higher than 90%, whatever the strain tested. Such a schizont-enriched culture can be very usefull for biochemical or molecular analysis [11]. This technique, when performed under sterile conditions and in a 37°C incubator, allowed a highly efficient synchronization of *P. falciparum *from in vitro cultures and rapid recovery of a highly synchronized parasite population. Moreover, it can be used to determine the point of action of a potential antimalarial drug [9].

**Table 1:** Enrichment of *P. falciparum *from in vitro cultures (comparison between Plasmion^®^, a gelatine solution, and magnetic columns)

	Parasitaemia (%)^a^		
Strain	Initial^c^	Plasmion^®^	Magnetic columns

FcB1 (K+)^b^	2.2	14.8 (6.7)^c^	98 (44)
FcM29 (K-)	2.8 (Fig. A)	3.4 (1.2).	96 (34) (Fig. B)
F32 (K+)	3.1	13 (4.2)	91 (29)
W2 (K+)	2.1	11 (5.2)	94 (44)

a: parasitaemia was evaluated by visual estimation and counting of at least 2,000 erythrocytes

b: presence (K+) or absence (K-) of knobs on erythrocyte membrane

c: in brackets, relative enrichment of the technique (parasitaemia after enrichment/initial parasitaemia)

### Concentration of *P. falciparum *gametocytes

As *P. falciparum *gametocytes also contained paramagnetic iron, it was of interest to test the ability of the MACS^® ^columns to purify them, either from in vitro cultures or from the peripheral blood of *Plasmodium *infected patients. Moreover, as gametocytes are not available in large amounts in blood or in culture, a highly purified population should be of interest for biochemical and/or molecular purposes. The use of MACS^® ^columns to improve the synchronous production of *P. falciparum *gametocytes in vitro has already been reported, but without any indication of the yield [12]. When tested on in vitro gametocyte culture, enrichment at a very high yield (enrichment rate higher than 100×) was obtained, whatever the gametocyte stage (II to IV) tested (see Table 2 and Figure 1E). Given this result, it was of interest to evaluate the efficiency of MACS^® ^columns for gametocyte enrichment in the field. With infected peripheral blood we obtained a relative enrichment of gametocytes ranging from 43 to 85 (Table 2 and Figures 1C and 1D). The results presented here only concerns three different patients, but a very high enrichment (at least more than 40 fold) was obtained with all the other patients tested. For *P. falciparum *infected blood, MACS^® ^columns enabled us to separate trophozoites (which did not contained haemozoin and were found in the first eluent) and gametocytes from the same donor with a very high reliability. Less than one parasite stage mismatch/1,000. This result allowed unambiguous biological or molecular studies on separate stages of the parasite from the same patient. In *P. falciparum *infection, the peripheral blood contains only trophozoites and the gametocyte stages IV and V, while schizonts and early gametocyte stages (I and II) are sequestered [13].

**Table 2:** Enrichment of gametocytes obtained with *P. falciparum *from infected blood from patients and in vitro cultures

	Initial^a^	Eluent^b^	Final count^c^	Enrichment rate^d^
Patients blood^e^	21	2	1120	53
	6	1	514	85
	19 (Fig. C)	1	819 (Fig. D)	43
In vitro culture	0.8 ± 0.03%^f^	< 0.01	91%	114
Stage II–III	0.9 ± 0.1%	< 0.01	88%	98
In vitro culture Stage IV–V	0.4 ± 0.02%	< 0.01	95%	237
	0.8 ± 0.1%	< 0.01	92%	115
	0.7% ± 0.1%	< 0.01	94% (Fig. 1-E)	134

a: calibrated thick films (5 μL), gametocyte count

b: calibrated thick films (5 μL), gametocyte count after elution from the columns

c: Calibrated thick films (5 μL), gametocyte count on the retained blood

d: calculated as ratio of final/initial counts

e: estimated on three patients with presence of gametocytes

f: estimated on thin blood smears (at least 10,000 red blood cells were counted).

### Non-*falciparum *purification

Peripheral blood containing non-*falciparum Plasmodium *was also tested for enrichment with MACS^®^. Whatever the species tested, the peripheral blood was enriched in erythrocytic stages (Table 3 and Figures 1F to 1H). Because in non-*falciparum *malaria all stages were in the blood circulation, the enrichment concerned all haemozoin-containing parasites (namely schizonts, Figure 1H and gametocytes Figure 1F) and led to high levels of enrichment (Table 3).

**Table 3:** Enrichment of non-*falciparum *parasitized erythrocytes

		Parasitaemia (%)	
Species		Initial:	After magnetic columns

*P. malariae*^a^	Trophozoites and schizonts	< 0.1	12
		0.3 (Fig. G)	17 (Fig. H)^b^
*P. ovale*	Trophozoites and schizonts	< 0.1	15
	Trophozoites schizonts and gametocytes	<0.01 and presence of gametocytes	6, and numerous gametocytes (Fig. F)
P. vivax	Trophozoites and schizonts	< 0.1	16

a: species and mono-infestation were confirmed by PCR

b: enrichment was performed 36 hours after blood collection

This is the first time that an easy-to-use and fast method has been described for human non-falciparum *Plasmodium *enrichment. The MACS^® ^columns, in the field, could be an efficient way to concentrate parasites and could associate, in one step, the advantages of thin smears and thick films with species diagnosis and enrichment. In research laboratories, the purification of non-*falciparum Plasmodium *in large enough amounts to work on specific pathways or to improve in vitro cultures [14] could also be facilitated by this material.

In conclusion, the simple one-step MACS^® ^columns extended here to all the human *Plasmodium *species allowed large-scale concentration and purification of different parasite stages, with emphasis on gametocytes from *P. falciparum *from both in vitro cultures and from *Plasmodium*-infected peripheral blood. This method offers opportunities to extend knowledge on the biology of *Plasmodium sp*.

## Authors' contributions

CR: Initiated falciparum purification, realized the experimental conditions and contributed to the preparation of the manuscript. AB: developed of the protocols and carried out most parts of the laboratory experiments for gametocyte carrier experiments, and contributed to the preparation of the manuscript. SC: maintained in vitro culture, produced gametocytes, concentrated parasites and helped in analysing results. KR,: was implicated in elaboration of the experimental conditions, contribution to the preparation of the manuscript. IM: was responsible in Cameroon for supervision of IRD/OCEAC laboratory work and protocol for gametocyte carrier recruitment. DP: assisted in redaction. FN: Head of the laboratory, discussion and assisted in redaction. FBV, initiated the falciparum gametocyte production, discussion concerning redaction and contributed to the preparation of the manuscript. AV: Redaction, non-falciparum purification, falciparum gametocytes purification, pictures. All authors read and approved the final manuscript.
